# Association of road traffic noise exposure with dementia or cognitive impairment – A systematic review of longitudinal cohort studies

**DOI:** 10.1371/journal.pgph.0006139

**Published:** 2026-03-18

**Authors:** Emil Basil Scaria, Nisha Dhanda

**Affiliations:** 1 Department of Applied Health Sciences, College of Medicine and Health, University of Birmingham, Dubai, United Arab Emirates; 2 Department of Applied Health Sciences, College of Medicine and Health, University of Birmingham, Edgbaston, United Kingdom; National Center for Chronic and Noncommunicable Disease Control and Prevention, Chinese Center for Disease Control and Prevention, CHINA

## Abstract

Road traffic noise is a major public health concern associated with cardiometabolic outcomes, sleep disturbances, noise annoyance, and cognitive effects. Dementia poses a significant health and socioeconomic burden. While previous reviews have examined environmental noise broadly, few have synthesised longitudinal evidence on road traffic noise and dementia or cognitive impairment. This review evaluates this association using clearly defined inclusion criteria focused on cohort study designs. MEDLINE, EMBASE, CINAHL Plus and GreenFile were searched for studies on road traffic noise exposure and the risk of dementia or cognitive impairment among adults from inception to July 2025 without restrictions on setting or geographical location. Longitudinal cohort studies were identified using strict inclusion and exclusion criteria. Two independent reviewers conducted screening, data extraction, and quality assessment. A narrative synthesis was conducted. 3296 studies were retrieved from the searches, of which 3264 were excluded and 32 underwent full text screening. 8 studies were narratively synthesised. Risk of bias assessment using the ROBINS-E tool indicated that most studies were judged to have ‘some concerns’, with one study assessed as high risk and one as low risk of bias. The findings suggest that adults exposed to high levels of road traffic noise, particularly >50 dB, compared to those exposed to lower levels, are at higher risk of developing dementia or cognitive impairment. Current evidence from longitudinal cohort studies suggests a modest, directionally consistent association between road traffic noise and dementia or cognitive impairment, though effect sizes were generally small and often not statistically significant. While heterogeneity in methods precluded meta-analysis, convergence of findings across large cohorts supports further investigation using robust longitudinal designs. From a public health perspective, mitigating night-time traffic noise may offer co-benefits for cognitive health, sleep, and cardiovascular outcomes, and should be considered in urban planning and noise regulation.

## 1 Introduction

Noise is an acoustic phenomenon of excessive or loud sound [1]. It is also referred to as noise pollution, which is characterised by environmental noise that is disruptive and disturbing [2]. Chronic exposure to excessive noise has been shown to have detrimental effects on human health [3,4]. Environmental noise, primarily from anthropogenic sources such as road, rail, air traffic, and industrial activity, is a key contributor to noise pollution and also has ecological implications. In recent years, research has shed light on the impact of noise pollution on human health, particularly among children and older adults, demonstrating associations with a range of adverse outcomes including cognitive and sleep disorders, mental health issues, cardiovascular diseases, and metabolic dysfunction [5,6]. Road traffic noise is a leading environmental pollutant and a growing public health concern. It is measured or modelled as the A-weighted equivalent continuous sound level (Leq) generated by vehicular sources including cars, heavy goods vehicles, and motorcycles which is measured or modelled at residential façades [7].The World Health Organization’s Burden of Disease from Environmental Noise report (2011) quantified over one million healthy life years lost annually in Europe due to environmental noise exposure, predominantly from road traffic sources [8]. In England, it is estimated 97,000 DALYs are lost due to road traffic noise [9].At the same time, dementia represents an urgent global health challenge. It is a neurocognitive disorder that impairs memory, executive function, and other cognitive domains, making routine activities challenging [10]. Globally, dementia affects an estimated 55 million individuals, with projections reaching 78 million by 2030 [11,12]. The condition develops gradually over many years, with symptoms beginning up to 10–12 years before clinical onset [13,14].Cognitive abilities decline progressively, leading to neuropsychiatric symptoms such as confusion, anxiety, depression, and apathy [15,16]. As the disease advances, individuals struggle with daily activities and face a heightened risk for debilitating conditions such as urinary incontinence and hip fractures [17,18], which significantly affects quality of life and increases dependence on nursing care [19,20].

Dementia arises from a complex interplay of multiple factors, including diet, lifestyle, physical activity, sleep, genetics, and cardiovascular disease [21,22] Advancing age is the primary risk factor for dementia [23,24], with women being at a slightly higher risk than men [25]. Environmental noise exposure has been found to be higher for people of lower socioeconomic status [26,27], and low socioeconomic status has been associated with an increased risk for developing dementia [28]. Residential areas with higher levels of noise, such as high-rise buildings in urban areas, can also contribute to an increased risk of dementia [29]. Social isolation, often a result of hearing loss caused by noise exposure [30], is another significant factor which has been linked to a higher likelihood of dementia [31].

While cognitive tests, psychiatric evaluations, and brain imaging are used to diagnose dementia, there is currently no cure for the condition. Pharmacological drugs are used to manage cognitive decline, while lifestyle changes and occupational therapies improve quality of life [32]. It is important to distinguish dementia from cognitive decline, which refers to reductions in performance in specific cognitive domains (e.g., memory and attention), whereas dementia denotes a clinical syndrome of multi-domain impairment that interferes with autonomy [33]. According to a 2019 study [34], the median survival for patients with dementia is 5.2 years, leading to significant public health resources being spent on managing the condition, providing nursing care, and treating comorbidities. The global annual cost of dementia, which included direct medical costs, direct social sector costs, and costs of informal care, was estimated at USD 1313.4 billion in 2019 [35].

Environmental noise contributes substantially to population health burden through well-established pathways including sleep disturbance and noise annoyance. The WHO Environmental Noise Guidelines identify sleep disruption as one of the most robust and consistently observed effects of road traffic noise, with downstream consequences for cardiometabolic and mental health. Noise annoyance, a stress-related response reflecting chronic environmental strain, has also been associated with chronic stress and vascular risk [36]. These pathways may plausibly contribute to neurodegenerative processes over time. Emerging evidence suggests that traffic noise exposure initiates a cascade of physiological responses that may contribute to neurodegenerative processes. Chronic exposure to elevated A-weighted Leq activates the hypothalamic–pituitary–adrenal (HPA) axis, resulting in sustained cortisol release and oxidative stress [37–39]. Animal studies have shown that both air and noise pollution cause neurodegenerative changes that lead to neuroinflammation and oxidative stress [40–42]. Noise-induced sleep fragmentation further exacerbates microglial activation and blood–brain barrier permeability, promoting neuroinflammation and accumulation of β-amyloid and tau proteins [43,44].

However, accurately measuring road traffic noise is challenging, as it is impractical to measure the noise emitted by each individual vehicle and measuring personal exposure is intrusive. The World Health Organization recommends using standard indices that measure road traffic noise using Leq, provides an average value for the total sound produced in an area during a specific period [45,46]. The Leq value over an entire day can be expressed as Lden, which includes penalties for evening and night-time noise. Leq during night-time may also be expressed as Lnight. The WHO guidelines recommend that road traffic noise (Lden) should remain below 53 dB and road traffic noise at night (Lnight) should remain below 45 dB to prevent adverse health outcomes [47]. Understanding the potential link between traffic noise and dementia is therefore critical for informing urban planning, transport infrastructure design, and noise mitigation policies, which could yield co-benefits for cardiovascular, mental, and cognitive health.

Previous research has examined the impact of road traffic noise on cognitive performance and dementia using both cross-sectional [48] and cohort [49,50] studies. However, these studies have yielded inconclusive results and been complicated by confounding factors and mediators. Although there is evidence linking road traffic noise exposure to hearing loss and hearing loss to dementia, there is insufficient conclusive evidence linking road traffic noise exposure to dementia directly. A longitudinal ecological time-series study suggests that short-term exposure to traffic noise may exacerbate symptoms and increase hospital admission rates for dementia [51]. Additional research has explored the relationship between exposure to both air pollution and noise with dementia. A large cohort study conducted in Canada found that living near high-traffic roads increased the incidence of dementia, which could be attributed to exposure to air or noise pollution [52]. Similarly, a cross-sectional analysis of participants aged 50–80 in the Heinz Nixdorf Recall study found that long-term exposure to air pollution and traffic noise was associated with mild cognitive impairment [48], with the association between traffic noise and global cognitive scores only seen with high exposure to air pollution [53]. A study in South Korea observed 11% higher odds of cognitive decline in people who reported exposure to noise pollution in their residential area [54].Despite growing interest in noise-related cognitive effects, most existing studies and reviews have relied on mixed sources of noise or cross-sectional designs, limiting causal interpretation. Only a few systematic reviews have examined the relationship between road traffic noise exposure and the risk of cognitive impairment or dementia in adults [55–57]. One review had multiple outcomes, which may have affected the quality of studies related to dementia and cognition. Additionally, it was commissioned by the UK Department for the Environment, Food and Rural Affairs, and therefore had a political agenda limited to the UK context [55]. Another review noted that the studies included had low methodological quality, which prevented adequate pooling of results [56]. A third study conducted a meta-analysis to investigate the dose-dependent relationship between noise exposure and dementia ris;however, the inclusion of case-control and cross-sectional studies limited assessment of temporality and introduced potential recall and selection bias [57]. Collectively, these limitations highlight the need for a systematic review focusing specifically on longitudinal cohort studies assessing the association between chronic exposure to road traffic noise and the risk of dementia or cognitive impairment.

This systematic review aims to assess whether long-term exposure to road traffic noise (Exposure), compared to lower or no exposure (Comparator), is associated with increased risk of dementia or cognitive impairment, including all-cause dementia and dementia subtypes such as Alzheimer’s disease, (Outcome) in adult populations (Population), using evidence from longitudinal cohort studies, and to synthesise the findings narratively. A quantitative meta-analysis was planned but was not conducted due to substantial heterogeneity in study methods and outcomes.

## 2 Methods

The review was conducted following the Preferred Reporting Items for Systematic Reviews and Meta-analyses (PRISMA) guidelines [58]. The completed checklist is provided in S1 Checklist PRISMA Checklist.

### 2.1 Search strategy

A scoping search and assessment of previous systematic reviews were carried out and used to design a search strategy. Systematic searches were carried out using tailored strategies on four databases - MEDLINE, EMBASE, CINAHL Plus and GreenFile in July 2025.

MEDLINE and EMBASE were searched as they are general bibliographic databases with broad coverage of biomedical and scientific literature. CINAHL Plus, with its extensive nursing and allied-health journal indexing, was searched to capture relevant clinical and epidemiologic studies; and GreenFILE was included as a dedicated environmental database covering different types of pollution and its human impact. A search for grey literature was conducted on BASE (Bielefeld Academic Search Engine) using relevant keywords such as ‘road traffic noise’ and ‘dementia’. This search included academic theses, technical reports, conference papers, and preprints. No language, date, or publication status restrictions were applied. The same inclusion and exclusion criteria used for peer-reviewed studies were applied to all grey literature sources. Of 43 records retrieved, none met the eligibility criteria or contributed new studies beyond those already captured in the primary database searches.

The search strategy included relevant search terms and MeSH headings. It was broad and included free text terms and MeSH headings related to traffic noise, noise pollution, dementia, cognitive decline, and cognitive impairment. They were combined appropriately using Boolean operators (AND, OR, NOT). Searches were not limited by time or language. The databases were searched from inception to July 2025. The strategy used for each database is available in S1 Text. The reference lists of published reviews and included studies were also searched to identify eligible studies. Full text articles for all studies were obtained using University of Birmingham library resources or through open access journals.

### 2.2 Study selection

A population-exposure-comparator-outcomes-study design (PECOS) was used to create the inclusion and exclusion criteria (see Table 1).

**Table 1:** Inclusion and exclusion criteria for study eligibility.

Inclusion Criteria	Exclusion Criteria
Prospective and retrospective cohort studies.	Randomised controlled trials, cluster randomised controlled trials, controlled before-and-after studies, non-randomised studies, case-control studies, cross-sectional studies and case series or reports.
Human studies	Animal studies
Studies including adults from the general population.	Studies including only children below the age of 18 years.
Studies with exposures related to traffic noise.	Noise related exposures not related to traffic noise.
Studies with traffic noise measured or modelled objectively.	Studies with traffic noise measured subjectively.
Studies with dementia or cognitive impairment as outcome.	Studies with outcomes unrelated to dementia or cognitive impairment.
Studies using any standard, internationally accepted diagnostic criteria like DSM-5 [10].	Studies not mentioning diagnostic criteria for outcome measurement.
Any mention of dementia, cognitive impairment, Alzheimer’s Disease, or neurocognitive disorders as outcomes due to traffic noise exposure.	Studies that only have cognitive tests as outcomes.
No language restrictions.	Studies specifically focused on individuals with manifest or diagnosed hearing impairment.
No timing restrictions	
No restrictions based on publication status	
No restrictions based on setting or geographical location.	

The eligibility criteria used to select studies for inclusion are summarised in Table 1. Additional clarifications for each domain are outlined below.

#### 2.2.1 Study design.

Prospective and retrospective cohort studies were considered for inclusion. Dementia is a disease of insidious onset and may be difficult to detect without long duration of follow-up. As there are no existing systematic reviews that focus solely on longitudinal study designs to explore this association and account for the temporal nature of this relationship, we have chosen to exclude other types. Randomised controlled trials and cluster randomised controlled trials have been excluded as high levels of noise exposure can cause adverse health outcomes, and randomisation to long-term interventions exposing participants to noise poses ethical concerns. Cross‐sectional studies were excluded because they cannot establish the temporal sequence between traffic noise exposure and subsequent onset of dementia, leaving findings vulnerable to reverse‐causation and survivorship biases. In addition, single‐time‐point assessments of both exposure and outcome are prone to bias, as participants with early, subclinical cognitive impairment may selectively relocate to quieter environments or be less likely to participate in noise surveys, thereby distorting true associations between exposure and outcome. By restricting our review to longitudinal cohort studies, we ensure that noise exposure is measured prior to disease onset, bolster causal inference through clear temporality, and minimise bias from unmeasured confounders and selective participation.

#### 2.2.2 Population.

Studies that only included children (below 18 years) were excluded as the outcome of interest is not expected in children. Dementia is a longstanding disease that develops over a span of 20–30 years with symptoms appearing commonly in the middle-aged or senile population [59]. Furthermore, scoping and literature reviews revealed that most primary studies focused on adults aged above 40 years. Studies including both children and adults were excluded as they may be prone to high levels of bias. Animal studies were excluded.

#### 2.2.3 Exposure.

Only studies using objective measurements or validated modelling of road traffic noise were included. This decision was made to minimise exposure misclassification, which is more likely in studies using subjective or self-reported noise levels. Studies assessing noise from non-road sources (e.g., industrial, occupational, or construction noise) were excluded to isolate the specific effects of road traffic noise. While other transportation noise sources such as aircraft and railway noise may also affect cognitive health, this review focused exclusively on road traffic noise to reduce exposure heterogeneity and reflect its dominant contribution to environmental noise burden in urban settings.

#### 2.2.4 Comparator.

Comparators were defined as groups with low or no road traffic noise exposure. This allowed for clear contrast with higher exposure groups and enabled relative risk estimation across exposure levels.

#### 2.2.5 Outcomes.

The primary outcomes were incident dementia or cognitive impairment. Studies were included if they assessed all-cause dementia or specific subtypes (e.g., Alzheimer’s disease, vascular dementia), as long as diagnosis was based on internationally accepted criteria such as Diagnostic and Statistical Manual of Mental Disorders, Fifth Edition (DSM-5) [10]. Studies that had only had cognitive tests as outcomes and studies focusing specifically on individuals with diagnosed or manifest hearing impairment were excluded to reduce misclassification and improve comparability.

#### 2.2.6 Timing.

There were no restrictions based on timing.

#### 2.2.7 Setting.

There were no restrictions based on the geographical or environmental setting of the study, such as country income level, urban versus rural location or healthcare system context.

#### 2.2.8 Language.

There were no restrictions based on language of publication.

#### 2.2.9 Publication.

There were no restrictions based on the publication date, with both published and unpublished studies (e.g., from grey literature databases) considered, provided full-text and sufficient methodological detail were available for risk of bias assessment. Retrieved search results included studies from inception (1946 for MEDLINE; 1974 for Embase) to July 2025.

### 2.3 Screening

All studies were imported into EndNote 20.2.1, a reference management software by Microsoft [60], and duplicates were removed. They were uploaded into Covidence, a systematic review management software, which was used to screen abstracts and full texts, as well as resolve disagreements [61]. Title and abstract screening were performed by two independent reviewers (ES and ND) and articles that met the inclusion criteria were selected. Disagreements were resolved thorough discussion by ES and ND. The full texts of studies that were selected after title and abstract screening were screened independently by two reviewers (ES and ND) for inclusion in the review using Covidence. Disagreements were resolved through discussion and review by ES and ND.

### 2.4 Data extraction

A data extraction form was used to extract relevant information from the included studies. The form created using Microsoft Excel has been provided (S2 Text). Data extraction was conducted by two reviewers (ES and ND). The form was piloted by one reviewer (ES) on two studies before further extraction was undertaken. A data extraction table was created to report the information extracted and help with comparison of extracted data. General information for each study such as title, authors, year of publication, study design, and location were extracted along with data related to population, exposure, outcome, and comparators. This included data related to noise source, exposure levels, measurement method, type of dementia, and diagnostic criteria, statistical methods, unadjusted and adjusted risk estimates such as hazard ratios (HR), 95% confidence intervals, p-values, confounders and covariates. Items that were used to conduct quality assessment were also extracted at this stage.

### 2.5 Outcomes

The primary outcomes were dementia or cognitive impairment. Studies were required to assess these outcomes using internationally accepted diagnostic criteria. Dementia was typically identified through either clinical diagnosis using standard, internationally accepted diagnostic criteria for both outcomes such as DSM-5 [10], DSM-4 [62] and International Classification of Diseases – 10^th^ Revision (ICD 10) [63,64] codes, or through validated registry-based coding systems. Some studies specified dementia subtypes, such as Alzheimer’s disease or vascular dementia, while others reported all-cause dementia. Cognitive impairment was defined based on global cognitive scores or neuropsychological testing, with thresholds (e.g., ≥ 1.5 SD below age norms) applied to define impairment. A small number of studies included both dementia and milder forms of cognitive decline as separate outcomes. Details on the specific criteria and assessment methods were extracted when multiple tools were used. This enabled evaluation of consistency and comparability across studies. Further details on diagnostic criteria and outcome definitions are summarised in Table 2 and Section 3.2.3.

**Table 2:** Characteristics of the studies included in the review.

Author	Year	Title	Age	Study design	Total n at baseline	Total n in analysis	Reasons for missing participants	Gender (M/F)	Exposure	Noise indicator and exposure category	Outcome	Time points	Average follow-up	Country	Effect estimate
Andersson et al. [68]	2018	Road traffic noise, air pollution, and risk of dementia– results from the Betula project	55-85 years	Cohort	1721	1721	Not applicable	57%/43%	Road traffic noise	Leq 24h > 55 dB vs < 55 dB	Dementia	1993-2010	7 years	Sweden	HR = 0.95 (0.57–1.57)
Cantuaria et al. [69]	2021	Residential exposure to transportation noise in Denmark and incidence of dementia: national cohort study	≥60 years	Cohort	1938994	1938994	Not applicable	47%/53%	Road traffic noise	Lnightmax >60 dB vs < 40 dB; Lnightmin >50 dB vs < 40 dB	Dementia	2004-2017	8.5 years	Denmark	Lnightmax: HR = 1.12 (1.10–1.15); Lnightmin: HR = 1.05 (1.01–1.09)
Carey et al. [70]	2018	Are noise and air pollution related to the incidence of dementia? A cohort study in London, England	50-79 years	Cohort	139718	130978	Existing dementia, living in care homes, patients without IMD^a^.	50%/50%	Road traffic noise	Lnight >53.8 dB vs < 49.4 dB	Dementia	2005-2013	6.9 years	United Kingdom	HR = 1.09 (0.95–1.25)
Ogurtsova et al. [71]	2023	Association of long-term air pollution and ambient noise with cognitive decline in the Heinz Nixdorf Recall study	50-85 years	Cohort	4184	2554	Missing covariates, incomplete exposure assessment, dementia diagnosis.	53%/47%	Road traffic noise	Mean noise level per 10 dB increase	Cognitive Decline	2006-2015	10 years	Germany	β = 0.052 (−0.05–0.06)
Yu et al. [72]	2020	Traffic-Related Noise Exposure and Late-life Dementia and Cognitive Impairment in Mexican-Americans	>60 years	Cohort	1789	1612	Missing exposures, dementia diagnosis, no baseline visit, no follow-up.	42%/58%	Road traffic noise	Lnight ≥55 dB vs < 55 dB	Dementia/CIND^b^	1998-2007	6.5 years	United States of America	HR = 1.16 (0.80–1.68)
Havyarimana et al. [73]	2025	Long-term road traffic and railway noise exposure and risk of dementia: UK Biobank cohort study	40-69 years	Cohort	502000	467905	Missing baseline covariates, missing exposure data, prevalent dementia at baseline	48%/52%	Road traffic and railway noise	Lden ≥60 dB vs < 50 dB	Dementia	2006-2021	11 years	United Kingdom	HR = 1.03 (0.84–1.26)
Tuffier et al. [74]	2024	Long-term residential road traffic noise exposure and risk of dementia: the Danish Nurse Cohort	>44 years	Cohort	28731	25233	Prevalent dementia, missing exposure data, missing covariates	0/100%	Road traffic noise	Lden per IQR increase	Dementia	1993-2020	15.4 years	Denmark	HR = 1.02 (0.93–1.11)
Wu et al. [75]	2024	Long-term exposure to transportation noise in relation to global cognitive decline and cognitive impairment: Results from a Swedish longitudinal cohort	>60 years	Cohort	3363	2594	Missing information on cognition score or cognitive impairment, dementia or CIND diagnosis, intellectual disability, schizophrenia diagnosis.	39%/61%	Road traffic noise	Lden per 10 dB increase	CIND	2001-2019	16 years	Sweden	HR = 0.98 (0.82–1.16)

^a^IMD – Index of Multiple Deprivation.

^b^CIND – Cognitive impairment with no dementia.

### 2.6 Quality assessment

The risk of bias was evaluated using the Risk of Bias In Non-randomised Studies–of Exposure (ROBINS-E) tool [65]. It is a structured tool developed at the University of Bristol to assess the risk of bias in observational studies, especially for systematic reviews. It uses seven domains with signalling questions to thoroughly assess the risk of bias, and uses this to grade the overall risk of bias. There are some limitations to the tool such as its inability to distinguish between confounders and co-exposures, as well as the usage of a rating system that fails to differentiate between studies with single and multiple risks of bias [66]. However, there is no perfect tool to assess the quality of observational studies of exposure, and ROBINS-E was considered more comprehensive and specific for this review compared to conventional quality assessment tools. Quality assessment items were extracted at the data extraction stage and two independent reviewers (ES and ND) conducted this process. Disagreements were resolved by discussion and review by ES and ND. The tool was piloted on an included study to assess its suitability before further steps were undertaken..

### 2.7 Synthesis

A meta-analysis was planned to pool effect estimates from eligible cohort studies where sufficient homogeneity in exposure metrics, outcome definitions, and effect measures existed. However, the included studies demonstrated substantial heterogeneity in noise metrics (Lden, Lnight, Leq), exposure categorisation, outcome ascertainment (all-cause dementia, Alzheimer’s disease, cognitive decline), and covariate adjustment. These differences precluded a meaningful pooled quantitative estimate. Therefore, a narrative synthesis was undertaken. The narrative synthesis followed guidance outlined by Popay et al. [67], structured to explore study characteristics, sources of heterogeneity, and convergence of findings. Heterogeneity was assessed conceptually based on differences in noise metrics, outcome classification, and covariate adjustment, rather than statistically. These dimensions informed how findings were grouped and interpreted. Study-level risk of bias was taken into account during narrative synthesis. Patterns in effect estimates were interpreted in light of ROBINS-E assessments, with greater weight given to studies rated as low risk. No formal sensitivity analysis was undertaken, but heterogeneity across noise metrics, outcome definitions, and confounder adjustment was explicitly described. All extracted summary estimates are given in S1 Table.

#### 2.7.1 Narrative synthesis.

The included studies were synthesised narratively, detailing the findings and results in the studies. The results were tabulated to allow for comparison between the studies. The conclusions were summarised and overall themes or patterns in the inferences noted.

## 3 Results

### 3.1 Study screening and selection

**Fig 1:**
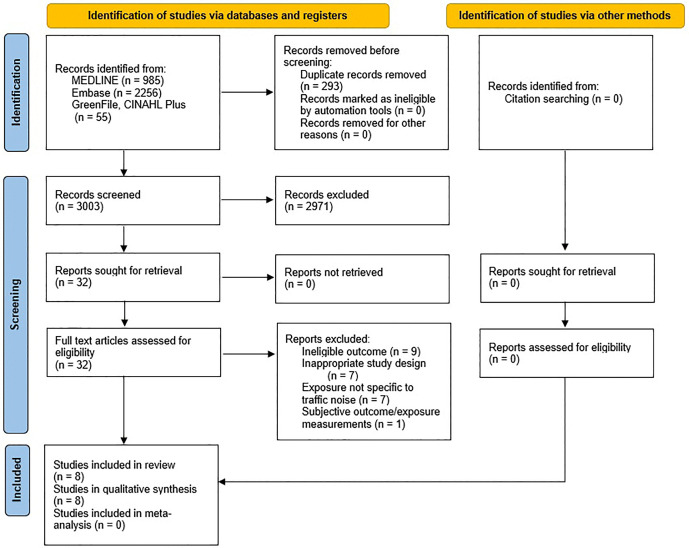
PRISMA [58] flowchart illustrating the study selection process.

The screening and selection process is summarised using a PRISMA diagram (Fig 1).

A comprehensive search of the four databases (MEDLINE, Embase, CINAHL Plus, GreenFile) identified 3296 records. No new papers were identified by searching the reference lists of systematic reviews or included studies. After removal of duplicates (n = 293), there were 3003 studies that underwent title and abstract screening. 2971 studies excluded based on their irrelevance to the review question and their eligibility as per the inclusion criteria. Full texts of the remaining 32 studies were obtained. All the studies were in English and did not require translation. The inclusion and exclusion criteria were applied and resulted in 24 studies being excluded.

8 studies [68–75] were selected for narrative synthesis, and the full texts and supplementary material were obtained. All the included studies used a cohort study design to explore the association between road traffic noise exposure and risk of dementia or cognitive impairment.

### 3.2 Study characteristics

Study characteristics are summarised in Table 2. All the studies included adults over the age of 40. Three studies [69,72,75] included adults aged over 60 years. One study only included female participants [74]. The number of male and female participants in the other included studies was almost equal, except two studies. One study [72] had 58% female and 42% male participants, while the other [75] had 61% female and 39% male participants. Two studies [68,69] did not have any missing participants and analysis included all the participants in the cohort. The remaining studies had to exclude participants from the analysis for various reasons, as detailed in Table 2. However, these studies conducted a complete case analysis and included only participants with complete data on exposure, outcome, and covariates in their analyses. The studies included a total of 2,571,591 participants, of which the overwhelming majority (n = 1,938,994) were from one study [69].

#### 3.2.1 Study design.

All the studies were prospective or retrospective cohort studies. Six studies derived their population from larger cohort studies. Cantuaria et al. [69] used the Danish Civil Registration System, Carey et. al [70] used a primary care database (Clinical Practice Research Datalink) in the UK, Wu et al. [75] used the Swedish National study on Aging and Care in Kungsholmen (SNAC-K), and Havyarimana et al. used the UK Biobank. Tuffier et al. [74] used data from the Danish Nurse cohort, and the sample only included female nurses. The average follow-up durations were between 6.5 -16 years for the included studies. The US study [72] had an average follow-up duration of 6.5 years and conducted follow-ups every 12–15 months, while the study with longest follow-up duration had an average follow-up of 16 years with participants assessed every 3–6 years [75]. One study [68] contained information of follow-ups conducted every 5 years during the study period, while two others [69,70] used information from yearly follow-ups.

#### 3.2.2 Exposure.

All eight included studies estimated residential road traffic noise exposure using computer-based modelling linked to residential addresses. The models were generated using validated methods and established noise mapping software, in accordance with national or regional noise mapping guidelines. Noise exposure metrics varied with studies reporting equivalent continuous sound levels (Leq or LAeq 24h), day-evening-night levels (Lden), or night-time levels (Lnight).

Leq/LAeq represents the equivalent continuous sound pressure level over a defined time period, A-weighted to better reflect human hearing. Lden is a 24 hour average that adds penalties for noise during the evening and night [76], while Lnight specifically captures noise between 23:00 and 07:00, a period when participants are most likely to be at home and potentially vulnerable to noise-related sleep disturbance [70]. Exposure classification approaches varied. Six studies [68–70,72,73,75] categorised noise exposure, typically defining “high” exposure as ≥ 50–55 dB (Leq, Lden, or Lnight) and “low” exposure as < 50 dB. Category cut-offs were largely consistent across studies, although specific thresholds varied slightly depending on the noise metric used. Two of these studies [73,75] also analysed noise exposure as a continuous variable, enabling estimation of risk per 10 dB increment. Two studies [71,74] only analysed noise exposure as a continuous variable.

#### 3.2.3 Outcome.

Six studies [68–70,72–74] examined dementia incidence as their primary outcome. One study [71] assessed cognitive decline using global cognitive scores, and another study [75] assessed CIND. Ascertainment via routine health/administrative records.

Four studies [69,70,73,74] identified dementia from linked clinical and administrative data. This offered complete national coverage and consistent coding over long follow-up, supporting large case counts and narrow CIs. However, registry algorithms may under-ascertain early or atypical cases and offer limited clinical detail compared to follow-up clinical evaluations. The remaining four studies [68,71,72,75] established outcomes through repeated cognitive testing and clinical evaluations. These studies provide clinically rich, adjudicated outcomes and can capture earlier cognitive changes. However, the resource-intensive process limited the sample sizes and resulted in wider CIs.

The diagnosis of dementia is a clinical decision made based on internationally accepted criteria that include substantial cognitive decline in one or more cognitive domains along with interference in performing daily activities. Cognitive decline is a reduction in cognitive capabilities, often assessed using a cognitive score, and constitutes a major criterion in dementia. The outcomes were diagnosed using standard, internationally accepted diagnostic tools. Two studies [68,72] used DSM-4 [62] and DSM-5 [10] criteria while four others [69,70,73,74] used ICD-10 [63] criteria. The remaining studies [71,75] derived standardised global cognitive scores from neuropsychiatric tests and compared intra-individually at baseline and follow-up to assess decline in cognitive scores. Alzheimer’s disease and vascular dementia were the most commonly reported subtypes when examined separately.

#### 3.2.4 Confounders.

The variables adjusted for in each study varied slightly. Most studies adjusted for potential confounders such as age, education, air pollution, and socioeconomic status. Other variables, including physical activity, smoking, alcohol use, BMI, and comorbidities such as hypertension and diabetes, may function as mediators or modifiers in the relationship between noise exposure and dementia, and should be interpreted cautiously in this context.

**Fig 2:**
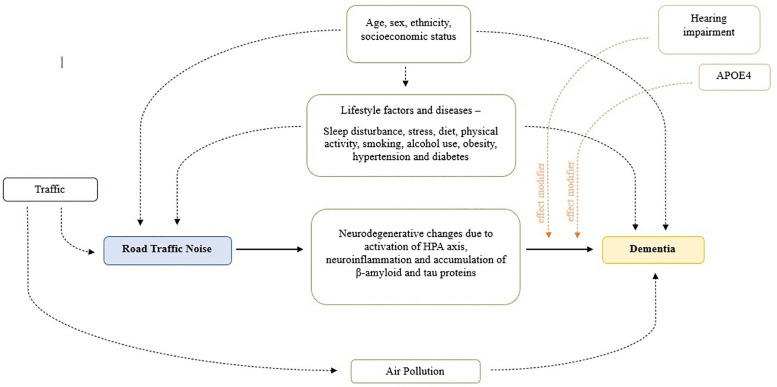
Conceptual model of potential pathways linking noise exposure with dementia.

Fig 2 presents a conceptual model illustrating potential causal pathways linking road traffic noise to dementia, as depicted in the existing literature, while also identifying potential confounders, mediators, and effect modifiers in this relationship.

#### 3.2.5 Context.

Seven studies were conducted within Europe (two each in Sweden, Denmark and the UK, and one in Germany), and one was from the United States of America. The research conducted in USA studied a population of Mexican-Americans in the country [72].

#### 3.2.6 Publication date.

All the studies were published between 2018–2025 but used data collected before 2021.

### 3.3 Quality Assessment

The methodological quality of the studies was assessed using the ROBINS-E tool. Studies were assessed over seven domains and graded for overall risk of bias as low, some concerns, high and very high risk as per the tool [65]. Six studies were deemed to pose ‘some concerns’ as there was a lack of clarity regarding the accuracy of results due to the presence of missing outcome, exposure, or covariate data. These studies used a complete case analysis to account for missing data. There was insufficient evidence available from the studies to determine whether the results were not affected by the incomplete data. One study [71] was deemed to be ‘high’ risk due to missing data in exposure, outcome and covariates, as well as concerns regarding outcome measurement. One study [69] was judged to have a ‘low’ risk of bias in all domains. The results for each domain and reasons for classifications are reported in Table 3.

**Table 3:** Risk of bias summary for each study with justification for overall rating using the ROBINS-E tool [65].

Study	Overall Risk of Bias	Domains with risk of bias^a^	Reasons
Andersson et al. (2018) [68]	Some concerns	D1	Some important variables such as neighbourhood-level factors (residence area), occupational status and type, ethnicity, and socioeconomic status were not controlled for.
Cantuaria et al. (2021) [69]	Low risk	None	None.
Carey et al. (2018) [70]	Some concerns	D5	6% of participants were excluded from the analysis due to missing exposure data.
Ogurtsova et al. (2023) [71]	High risk	D5	Missing exposure, outcome and covariate data that may have affected results despite a complete case analysis.
Yu et al. (2020) [72]	Some concerns	D5	Missing outcome data for 57 participants who were excluded from the analysis.
Havyarimana et al. (2025) [73]	Some concerns	D5	Missing exposure, outcome and covariate data that may have affected results despite a complete case analysis.
Tuffier et al. (2024) [74]	Some concerns	D1, D5	Some confounders not adjusted, and missing data in outcome.
Wu et al. (2024) [75]	Some concerns	D5	Missing exposure, outcome and covariate data that may have affected results despite a complete case analysis.

^a^The ROBINS-E tool consists of seven domains that gauge the risk of bias in different areas.

D1 – bias due to confounding.

D2 – bias arising from measurement of the exposure.

D3 – bias in selection of participants into the study (or into the analysis).

D4 – bias due to post-exposure interventions.

D5 – bias due to missing data.

D6 – bias arising from measurement of the outcome.

D7 – bias in selection of the reported result.

It was noted that all the studies had large sample sizes, did not use subjective noise measurement tools such as self-reported questionnaires or non-validated surveys, and offered clear diagnostic criteria for outcome assessment. The studies provided adequate information regarding participant characteristics, demographics, methods of exposure and outcome assessment, and statistical analysis. All the studies performed multi-variate analyses using a combination of different confounders that were identified as important. The studies were observed to have low to moderate risk of bias, with residual confounding and bias due to missing data being the only areas of concern. All studies adjusted for age, physical activity, and socioeconomic status. None adjusted for APOE ε4 genotype, which may act as an effect modifier by altering susceptibility to noise-related cognitive decline. Only Wu et al. adjusted for baseline hearing impairment.

### 3.4 Narrative synthesis

A narrative synthesis was completed to explore the themes and results across the eight included studies. Table 2 presents study characteristics alongside noise exposure contrasts and effect estimates, allowing for comparison of outcomes across exposure metrics. Table 4 complements this by detailing how each study measured outcomes, the modelling methods used to estimate noise exposure, and the covariates included in adjusted models. The studies varied in population size, geographic location, exposure metrics, and outcome definitions. All studies modelled noise exposure at the residential address, most often as Lden or Lnight, with cut-offs around 50–55 dB to define higher exposure. Five studies examined dementia incidence as a primary outcome [68–70,73,74], two examined cognitive impairment not meeting dementia criteria or cognitive decline [71,75], and one examined both dementia and cognitive impairment in a specific population [72]. A graphical display of the effect sizes from individual studies without pooling is provided in S1 Fig.

**Table 4:** Included studies with outcome, exposure, and covariate information.

Study	Outcome	Outcome measurement criteria/methods	Exposure measurement method	Covariates controlled
Andersson et al. (2018) [68]	Dementia	DSM-4 criteria^a^	Computer-generated model. Residential address of participants used to link noise exposure.	Age, nitrogen oxide levels, education, physical activity, smoking, sex, BMI, waist-hip ratio, alcohol, ApoE4, medical history of diabetes, hypertension, stroke
Cantuaria et al. (2021) [69]	Dementia	ICD-10 criteria^b^	Nordic prediction method using SoundPLAN software in Lnight.^c^	Age, sex, calendar year, civil status, income, region of origin, occupation, proportion of high-quality green space, income, basic education, who are single parents and with a criminal record, railway noise.
Carey et al. (2018) [70]	Dementia	ICD-10 criteria^d^	TRAffic Noise Exposure (TRANEX) model.	Age, gender, ethnicity, smoking, alcohol, BMI, IMD.
Ogurtsova et al. (2023) [71]	Cognitive Decline	Global Cognitive Score (GCS)^e^	Noise modelled according to the European Union Directive 2002/49/EC. Noise exposures assigned to participants using the maximum estimated noise value at each participant’s address	Age, sex, iSES, nSES, alcohol, BMI, diet, physical activity, smoking, cumulative smoking, environmental tobacco smoke exposure.
Yu et al. (2020) [72]	Dementia	DSM-5 criteria^f^	Federal Highway Administration (FHWA) Traffic Noise Model. Residential address of participants used to link noise exposure.	Age, gender, education, occupation during most of life, smoking, alcohol, physical activity, neighbourhood socioeconomic status, residential county, baseline Charlson index, baseline cognition, primary language.
Havyarimana et al. (2025) [73]	Dementia	ICD-10 criteria	Modelled using CNOSSOS-EU method (DEFRA) at 10 m façade height for 2010, with annual average Lden and Lnight values; assigned to baseline residential address via Ordnance Survey AddressBase.	Age, sex, ethnicity, Townsend deprivation index, education, employment, smoking, alcohol, BMI, physical activity, cardiovascular risk score, PM₂.₅, greenness, and railway noise.
Tuffier et al. (2024) [74]	Dementia	ICD-10 criteria	Modelled at all residential addresses using Nord2000 method; day, evening, and night penalties applied to produce Lden values at the most exposed façade.	Age, calendar year, smoking, alcohol, physical activity, waist circumference, BMI, marital status, education, income, PM₂.₅.
Wu et al. (2024) [75]	Cognitive impairment, no dementia (CIND)	Diagnosis based on a comprehensive neuropsychological test battery (episodic memory, language, perceptual speed, executive function, visuospatial ability) using the Graham approach. Impairment defined as ≥1.5 SD below the age-specific mean in any domain	Modelled using Nordic prediction method, using traffic volume, speed, vehicle type, road type, terrain/building screening, and calculated Lden at the most exposed façade for each address. 10-year time-weighted averages before baseline and 3-year moving averages during follow-up were derived.	Age, sex, education, birth year, baseline year; smoking, physical activity, neighbourhood household income, number of medications, hearing loss; 10-year average PM₂.₅, green space (NDVI), water space; and other transportation noise sources.

Abbreviations: DSM-4 = Diagnostic and Statistical Manual of Mental Disorders, Fourth Edition; dB = decibels; BMI = body mass index; ApoE4 = Apolipoprotein E4; ICD-10 = International Classification of Disease, Tenth Revision; Lnight = A-weighted, equivalent noise level at night; IMD = Indices of Multiple Deprivation; iSES = individual socioeconomic status; nSES = neighbourhood socioeconomic status; DSM-5 = Diagnostic and Statistical Manual of Mental Disorders, Fifth Edition.

^a^A specialist in geriatric psychiatry or medicine, made the diagnosis using DSM-4 criteria.

^b^Diagnostic information obtained from Danish National Patient Register or the Danish Psychiatric Central Register.

^c^Yearly noise was estimated from 1994-2017 for all addresses, and participants linked using residential addresses from the national health registry.

^d^Diagnosis was obtained from primary care records using Read codes for dementia within the Quality and Outcomes Framework (QOF), and postcodes used to link noise levels to participants.

^e^Neuropsychological tests were conducted and scores standardised. GCS was calculated as the sum of five age standardized scores of individual tests, and decline in GCS was calculated as difference between GCS at baseline and GCS at follow-up.

^f^The Modified Mini-Mental State Examination (3MSE) and a delayed word recall trial from the Spanish English Verbal Learning Test (SEVLT) for each patient was conducted at baseline and follow-up visits. Participants with low scores were referred for standard neuropsychological examination and the diagnosis confirmed with MRI scans

In interpreting the findings, it is important to consider the methodological strengths and limitations of the included studies, which provide context for the observed heterogeneity. The included studies varied in design features, exposure assessment, and outcome ascertainment, each contributing unique strengths and limitations. Andersson et al. (2018) conducted a clinical cohort study with dementia diagnoses confirmed by specialists and an average follow-up of seven years, ensuring near-complete capture of incident cases and minimal attrition. However, noise exposure was modelled only at baseline residential addresses, introducing potential misclassification due to unaccounted residential mobility. Cantuaría et al. (2021) leveraged a nationwide Danish cohort with registry-based dementia ascertainment and rich area-level covariates, producing highly precise hazard ratios and strong generalisability. In contrast, Carey et al. (2018) relied on primary care and mortality registry codes, which may have led to under-recording of dementia diagnoses. Ogurtsova et al. (2023) examined a single regional cohort using repeated neuropsychological testing, enhancing validity for cognitive outcomes but facing limited follow-up and possible selection bias. Yu et al. (2020) applied detailed noise modelling linked to geocoded addresses but was constrained by a small sample size, resulting in wide confidence intervals. Havyarimana et al. (2025) analysed the UK Biobank cohort, offering large sample size and long follow-up of approximately 11 years with comprehensive dementia ascertainment, though exposure was modelled only at baseline, again risking misclassification. Tuffier et al. (2024) provided a unique perspective through a nationwide female nurse cohort with detailed residential histories and time-varying exposure estimates, but generalisability was limited by its single-sex occupational sample, and recent exposures were imputed, potentially underestimating variability. Finally, Wu et al. (2024) combined validated neuropsychological assessments over 16 years with detailed noise modelling and extensive covariate adjustment, though its smaller sample size, infrequent follow-up intervals, and lack of dietary or genetic data were notable limitations.

Several studies did not find statistically significant associations for all-cause dementia, whereas others reported modest positive associations under specific conditions such as Alzheimer’s disease subtype or night-time exposure. Larger cohorts [50,74] generally produced narrower confidence intervals and more consistent risk estimates, while smaller studies yielded imprecise estimates. Differences in confounder adjustment were also evident: all studies controlled for key sociodemographic and lifestyle factors, and most included at least one air pollutant. However, psychosocial factors such as social isolation and mental health were unmeasured, and only Wu et al. [76] adjusted for baseline hearing impairment. Two studies [70,73] did not adjust for air pollution.

## 4 Discussion

This review found that higher exposure to road traffic noise was generally associated with a modest increase in dementia risk across longitudinal cohorts. While effect sizes were small, they may still have meaningful public health implications given the widespread nature of noise exposure. The association was more consistently observed for night-time noise exposure and in studies using large registry-based datasets. These findings support the need for environmental noise mitigation policies, particularly in densely populated urban settings. Importantly, in studies that adjusted for both noise and air pollution, the association between noise and dementia often attenuated but did not disappear after PM₂.₅ adjustment [50,75], suggesting both shared and independent pathways. A meta-analysis was initially planned but not conducted due to substantial heterogeneity in exposure metrics (e.g., Lden vs Lnight), exposure contrasts, and outcome definitions across studies. Several studies lacked the detail needed to harmonise or convert noise indicators, and attempting such transformations would introduce bias due to unverified assumptions. Consistent with best practice in environmental noise research, we present a structured narrative synthesis with stratified effect summaries. Dementia is a progressive disorder with an uncertain duration between noise exposure and onset. Nevertheless, research on other risk factors with similar mechanisms offers an estimate. Studies on known risk factors for dementia, such as hypertension and high cholesterol, suggest that dementia can manifest about 10 years after exposure [77–80]. Therefore, long-term studies with regular and complete follow-up assessments provide high-quality evidence. The studies in this review had an average follow-up duration of 6.5 to 16 years, which provide good evidence of association.

All studies in this review used computer-generated models to estimate noise exposure. While direct measurements can also be used, computer-enabled modelling is preferred for long-term monitoring and research studies. Sound level meters and noise dosimeters are validated devices that provide accurate measurements [46], but they are costly and limited in their geographical application. In contrast, modelled noise estimations can efficiently estimate exposure levels over large areas for extended periods [81]. However, these models rely on assumptions and can be inaccurate in capturing local variations. Similarly, dementia outcomes were ascertained through national health registries (ICD codes) or electronic health records, which may lack sensitivity for early-stage disease. Standardising diagnostic protocols and integrating cognitive testing batteries could improve comparability across future investigations.

The relationship between road traffic noise and dementia is complicated by the presence of confounders, such as exposure to air pollution, which has not been adequately studied. While some studies have adjusted for air pollution, there is little research on its actual impact on the relationship between noise exposure and dementia. Additionally, studies investigating the effects of air pollution and noise pollution independently are methodologically challenging due to their tendency to occur in combination, and it is unclear whether their combined risks are linear. In this context, it is essential to distinguish true confounders which are associated with both exposure and outcome, from mediators and effect modifiers. Age, sex, socioeconomic status, and air pollution are considered primary confounders, as they are plausibly associated with both noise exposure and dementia risk. Other factors such as hypertension, hearing impairment, or APOE ε4 genotype are established dementia risk factors but are not known to be associated with traffic noise exposure. These may therefore function as mediators or effect modifiers rather than confounders. Most included studies adjusted for major confounders, although adjustment for potential mediators was variable and should be interpreted accordingly. Certain potentially important mediators or modifiers such as social isolation and psychosocial factors were not adjusted for in any of the studies. Social isolation is linked to age [82] and hearing impairment [83], which are both associated with dementia independently, and may be part of a chain of causation leading to poor neurocognitive outcomes. A study published in 2023 found that social isolation increases the risk of dementia by 30% [31], and this risk was not modified by genetic factors [84]. This suggests that other factors, such as psychosocial factors, may also play a role in the development of dementia.

Differences in noise metrics, facade selection (most- vs least-exposed), and exposure time windows likely contribute to varied results. Outcome definitions also varied. Registry-based dementia captures later disease stages with high specificity but lower sensitivity, and cognitive decline and CIND may detect earlier changes but are not equivalent to dementia incidence.

### 4.1 Strengths of the review

This review critically assessed high-quality cohort studies examining the connection between exposure to road traffic noise and dementia. Following best practices in systematic review methodology, the review incorporated PRISMA guidelines and involved two researchers in the screening, data extraction, and risk of bias assessments. The review only included longitudinal studies, which are the most robust study design to explain this relationship, as randomised controlled trials would be highly unethical and inappropriate in this context. Previous reviews that included other study designs, such as cross-sectional studies, suffered from recall and selection bias, affecting the accuracy of their results.

### 4.2 Limitations of the review

While the search was extensive, only a few primary studies were available, which limited the number of studies included in the review and increased the heterogeneity between them.

Upon analysing the primary studies included in this review, it was observed that the studies assessed road noise exposure using modelled data, linking predicted noise levels in each area to the participants’ addresses. While cost-effective, this method is prone to misclassification bias as exposure may vary based on factors such as time spent at home, use of personal noise-protection equipment, and sound-proofing in construction. The exposure is usually modelled based on the loudest façade, and this may also result in an overestimation of the exposure [85]. Validation sub-studies using personal noise dosimeters are needed to quantify measurement error and refine dose–response estimates. Additionally, all except three studies [69,74,75] failed to collect complete residential history, which is an important essential determinant of exposure duration. Four studies [69,70,73,74] used primary care records and health registers, respectively, to obtain dementia diagnosis, which could have been prone to outcome misclassification. Underreporting and underdiagnosis of dementia is also an acute issue in primary care [86].

Exposure metric variability (24-hour Leq versus Lnight or Lden) limited direct comparability and precluded unified dose-response analyses. Although these modelled indicators are typically highly correlated since they are derived from standardised diurnal distributions, they may still differ in how they reflect health-related exposure windows. Leq captures overall daily exposure but may underrepresent nighttime peaks critical for sleep disturbance, whereas Lnight specifically addresses nocturnal exposure at the expense of missing daytime noise burdens. Lden applies a weighted 24-hour average with added penalties for evening and night-time noise to reflect increased sensitivity during these periods, but this aggregation can mask short-term peaks, rely on arbitrary penalty values, and reduce temporal specificity, potentially obscuring the distinct health impacts of daytime versus nocturnal exposure.

Six of the eight studies [70–75] had missing data, and all of them used a complete case analysis. This approach reduces the sample size and may lead to biased results because participants with missing data may differ from those with complete data. A better solution is to impute missing data, which would preserve data from participants with missing information and provide a less biased estimate. Complete case analysis remains a simpler method and can be reasonable when the proportion of missing data is small, as was the case in some cohorts. However, in larger datasets with complex exposure and outcome measures, imputation could improve precision and validity of effect estimates. Two studies [68,69] reported the same number of participants at baseline and in their main analyses, suggesting minimal or no missingness for variables used in the models. The possibility of bias due to unaccounted confounders is also a concern. None of the studies except Wu et al. [75] adjusted for baseline hearing impairment, a potential confounder that could bias associations if hearing loss both increases susceptibility to noise-induced stress and correlates with cognitive decline. Lastly, the results may not be applicable globally as the studies included were conducted in Europe or North America and only represent a small portion of the world’s population.

### 4.3 Implications and further research

An important caveat is the scarcity of robust longitudinal investigations examining traffic noise and neurodegeneration. Most existing studies employ cross‐sectional designs, which preclude causal inference and may be confounded by unmeasured factors. Further high-quality research is needed to explore the impact of road noise on cognitive health outcomes. It is suggested that symptoms of dementia occur long before diagnosis is made [87], and most large cohort studies with cognitive impairment or dementia as outcomes have insufficient follow-up periods for cognitive screening tests. Only a handful of cohort studies have followed participants over extended periods with repeated noise assessments and clinical endpoints, limiting our ability to characterise temporal relationships and dose–response dynamics. Future research should prioritize prospective designs with standardised exposure metrics and long‐term follow‐up to more definitively establish a link between chronic traffic noise and dementia development.

All included studies were conducted in high-income countries, predominantly in Europe and North America. Urban form, vehicle fleet composition, and noise modelling approaches differ substantially in low- and middle-income settings. Thus, the urgent need for prospective studies in Asia, Africa, and Latin America, where traffic mixtures and socioeconomic factors may modify exposure–response relationships. Pollution exposure is influenced by geography and is not consistent across all regions. Ethnicity is linked with an increased risk of dementia [88], and some ethnic groups such as African-Americans and Caribbean-Hispanics may be more susceptible to developing dementia than others [89,90]. Urbanisation and population growth have led to an expansion of road networks and increase in traffic [91], which exposes people living in densely populated areas and those without access to developed public transport systems to higher levels of road traffic noise [92]. Hence, low socioeconomic status is also associated with an increased exposure to traffic noise [93,94]. Vehicle honking, which adds 2–5 dB(A) to noise levels, is a significant contributor to road traffic noise [95,96] and can be influenced by cultural differences where honking is used as a form of non-verbal communication [97].

Although traffic noise has been linked to cognitive decline, none of the cohort studies in our review reported stratified analyses or examined potential effect modification by the APOE ε4 allele, the most significant genetic risk factor for dementia [98,99]. Future cohorts should collect biospecimens for genotyping and incorporate stratified analyses or interaction testing to determine whether noise-related dementia risk is modified by APOE status. Future studies must also harmonise noise metrics (e.g., prioritize Leq24h or Lden) and include audiometric assessments to control for hearing status. Dementia sub-types must be disaggregated, and consistent modelling strategies must be used to clarify independent effects of covariates.

## 5 Conclusion

In conclusion, existing evidence from longitudinal studies suggests a possible association between higher levels of road traffic noise and increased risk of dementia or cognitive impairment. While effect estimates were generally in a positive direction, most were modest and not statistically significant. Given the widespread nature of noise exposure, even small relative risks could be important at the population level. Future research should standardise noise‐exposure metrics, integrate APOE ε4 genotyping and hearing‐impairment adjustments, validate modeled exposures with personal dosimetry, and expand prospective cohorts to low- and middle-income settings.

From a public health perspective, interventions to reduce night-time transportation noise, through the installation of quiet façades, better insulation, and urban planning, are justified given potential neurological, cardiovascular, and sleep benefits. This may have far-reaching implications on the planning of transport infrastructure and development of policies to mitigate or prevent adverse health outcomes due to exposure to high levels of road traffic noise.

## Supporting information

S1 Checklist PRISMA Checklist. PRISMA 2020 checklist for reporting systematic reviews. From: Page MJ et al. The PRISMA 2020 statement: an updated guideline for reporting systematic reviews. BMJ. 2021;372:n71. Licensed under CC BY 4.0.
(DOCX)

S1 Text Full electronic search strategies used to identify eligible studies in MEDLINE (Ovid), Embase (Ovid), GreenFile, and CINAHL Plus, including search terms, limits, platforms, and dates of searches. (DOCX)

S2 Text Template used for extraction of study characteristics, exposure and outcome definitions, statistical methods, covariates, and effect estimates for included studies. (DOCX)

S1 Table Summary of exposure contrasts, effect estimates (hazard ratios or differences in standardized scores with 95% confidence intervals), and covariates included in progressively adjusted models for all studies included in the review. (DOCX)

S1 Fig Forest plot displaying study-specific hazard ratios for dementia associated with higher versus lower road traffic noise exposure across included cohort studies. The figure provides a visual comparison of effect estimates and 95% confidence intervals; results are shown without pooling due to heterogeneity in exposure metrics, outcome definitions, and analytical approaches.
(TIF)
