# Supplementary material for: Association of road traffic noise exposure with dementia or cognitive impairment – A systematic review of longitudinal cohort studies
Source: PLOS Glob Public Health. 2026 Mar 18;6(3):e0006139. doi: 10.1371/journal.pgph.0006139 (PMC12998818; doi:10.1371/journal.pgph.0006139)
Supplement: S1 Text — (DOCX) [file pgph.0006139.s002.docx]

**S1 Text**

**Search Strategy**

**Database: MEDLINE**

The literature search was conducted on MEDLINE using the Ovid platform on July 23, 2025. The search identified 985 records.

Ovid MEDLINE(R) ALL <1946 to July 23, 2025>

1 exp Noise, Transportation/ or exp Noise/ 28785

2 ((noise$ or sound$) adj pollution).ti,ab. 1273

3 ((environmental or traffic or city or urban) adj2 noise$).ti,ab. 3748

4 1 or 2 or 3 31058

5 exp Dementia/ or exp Frontotemporal Dementia/ or exp Dementia, Vascular/ 229541

6 exp Cognition Disorders/ or exp Cognitive Dysfunction/ 130284

7 exp Cognition Disorders/ or exp Cognition/ 327873

8 exp Alzheimer Disease/ 134979

9 exp Neurocognitive Disorders/ 346839

10 5 or 6 or 7 or 8 or 9 534786

11 (cognitive adj1 (impairment or deficit or decline or dysfunction$)).ti,ab. 146069

12 ((frontotemporal or vascular) adj dementia).ti,ab. 17731

13 (alzheimer or (alzheimer adj disease$)).ti,ab. 33003

14 ((cognitive or neurocognitive) adj disorder$).ti,ab. 11314

15 10 or 11 or 12 or 13 or 14 607269

16 4 and 15 1073

17 limit 16 to human 985

**Database: Embase**

The literature search was conducted on Embase using the Ovid platform on July 23, 2025. The search identified 2256 records.

Embase <1974 to 2025 July 23>

1 exp noise/ or exp traffic noise/ or exp noise pollution/ 175055

2 ((noise$ or sound$) adj pollution).ti,ab. 1500

3 ((environmental or traffic or city or urban) adj2 noise$).ti,ab. 4567

4 1 or 2 or 3 176051

5 exp dementia/ or exp frontotemporal dementia/ or exp senile dementia/ 515271

6 exp cognitive defect/ 720414

7 exp Alzheimer disease/ 287885

8 (cognitive adj1 (impairment or deficit or decline or dysfunction$)).ti,ab. 220741

9 ((frontotemporal or vascular) adj dementia).ti,ab. 26364

10 (alzheimer or (alzheimer adj disease$)).ti,ab. 44643

11 ((cognitive or neurogcognitive) adj disorder$).ti,ab. 10903

12 5 or 6 or 7 or 8 or 9 or 10 or 11 762256

13 4 and 12 2617

14 limit 13 to human 2256

**Database: EBSCOhost – GreenFile, CINAHL Plus**

The literature search was conducted on GreenFile and CINAHL Plus using the advanced search function on July 23, 2025. The search identified 55 records. The key words used in the search are as follows:

‘traffic noise pollution’ OR ‘road noise’ OR ‘traffic noise’ AND ‘dementia or alzheimers or cognitive impairment’ OR ‘cognitive decline’
