# Supplementary material for: Association of road traffic noise exposure with dementia or cognitive impairment – A systematic review of longitudinal cohort studies
Source: PLOS Glob Public Health. 2026 Mar 18;6(3):e0006139. doi: 10.1371/journal.pgph.0006139 (PMC12998818; doi:10.1371/journal.pgph.0006139)
Supplement: S2 Text — (DOCX) [file pgph.0006139.s003.docx]

**S2 Text**

**Sample Data Extraction Form**

Date:

Extracted by:

Study:

1. Authors:
2. Year of publication:
3. Title:
4. Study design:
5. Age group:
6. Males/Females:
7. Duration of study:
8. Country:
9. Total participants at baseline:
10. Total participants in analysis:
11. Reasons for missing participants:
12. Exposure source:
13. Exposure measurement method:
14. Comparator:
15. Follow-up duration:
16. Outcome:
17. Outcome diagnosis method:
18. Outcome diagnostic criteria:
19. Statistical method used:
20. Unadjusted result (95% CI):
21. Adjusted result (95% CI):
22. Covariates adjusted for:
23. Key conclusions:
