# Supplementary material for: Association of road traffic noise exposure with dementia or cognitive impairment – A systematic review of longitudinal cohort studies
Source: PLOS Glob Public Health. 2026 Mar 18;6(3):e0006139. doi: 10.1371/journal.pgph.0006139 (PMC12998818; doi:10.1371/journal.pgph.0006139)
Supplement: S1 Table — (DOCX) [file pgph.0006139.s004.docx]

**S1 Table**

**Effect sizes and covariates by each study included in the review.**

| **Study** | **Exposure/Comparator** | **Result** | **Adjusted for** |
| --- | --- | --- | --- |
| Andersson et al. (2018) (1) | Leq 24h ≥ 55 dB reference < 55 dB | HR = 0.99 (95% CI: 0.62-1.59) | Baseline age. |
|  |  | HR = 0.97 (95% CI:0.58-1.60) | Baseline age, sex education, physical activity, smoking, BMI, waist-hip ratio, alcohol, ApoE4. |
|  |  | HR = 0.95 (95% CI:0.57-1.57) | Baseline age, sex education, physical activity, smoking, BMI, waist-hip ratio, alcohol, ApoE4, baseline medical history of diabetes, hypertension, stroke. |
| Cantuaria et al. (2021) (2) | Lnightmax  <40 dB  40-45 dB  45-50 dB  50-55 dB  ≥60 dB | HR = 1 (Reference)  HR = 1.11 (95% CI: 1.08-1.13) HR = 1.13 (95% CI: 1.11-1.15) HR = 1.13 (95% CI: 1.11-1.16) HR = 1.12 (95% CI: 1.10-1.15) | Age, sex, calendar year, civil status, income, region of origin, occupational status, proportion of high-quality green space, area-level socioeconomic variables: percent population with low income, only basic education, unemployed, with manual labour, single parents and with a criminal record, and mutual road traffic and railway noise adjustment. |
|  | Lnightmin  <40 dB  40-45 dB  45-50 dB  ≥50 dB | HR = 1 (Reference)  HR = 1.09 (95% CI: 1.08-1.11) HR = 1.07 (95% CI: 1.05-1.10) HR = 1.05 (95% CI: 1.01-1.09) | Age, sex, calendar year, civil status, income, region of origin, occupational status, proportion of high-quality green space, area-level socioeconomic variables: percent population with low income, only basic education, unemployed, with manual labour, single parents and with a criminal record, and mutual road traffic and railway noise adjustment. |
| Carey et al. (2018) (3) | Lnight  0-49.4  >49.4-49.6  >49.6-50.3  >50.3-53.8  >53.8 | HR = 1 (Reference)  HR = 1.05 (95% CI: 0.91-1.20)  HR = 1.04 (95% CI: 0.90-1.19)  HR = 1.00 (95% CI: 0.87-1.15)  HR = 1.09 (95% CI: 0.95-1.25) | Age, gender, ethnicity, smoking, alcohol consumption, BMI & IMD. |
| Ogurtsova et al. (2023) (4) | For every 10 dB increase in noise | Difference in standardised score = 0.125 (95% CI: -0.421, 0.67) | Nil |
|  |  | Difference in standardised score = 0.073 (95% CI: -0.474, 0.62) | Age, sex, iSES and nSES. |
|  |  | Difference in standardised score = 0.052 (95% CI: -0.496, 0.6) | Age, sex, iSES, nSES, alcohol consumption, BMI, diet, physical activity, smoking status, cumulative smoking, and environmental tobacco smoke exposure. |
| Yu et al. (2020) (5) | Lnight ≥ 55 dB reference < 55 dB | HR = 1.14 (95% CI: 0.79, 1.64) | Baseline age, gender, years of education. |
|  |  | HR = 1.13 (0.79, 1.63) | Baseline age, gender, years of education, occupation. |
|  |  | HR = 1.16 (0.80, 1.67) | Baseline age, gender, years of education, occupation, smoking status, alcohol consumption status, physical activity level. |
|  |  | HR = 1.18 (0.82, 1.71) | Baseline age, gender, years of education, occupation during most of life, smoking status, alcohol consumption status, physical activity level, neighbourhood socioeconomic status indicator, residential county. |
|  |  | HR = 1.20 (0.83, 1.73) | Baseline age, gender, years of education, occupation during most of life, smoking status, alcohol consumption status, physical activity level, neighbourhood socioeconomic status indicator, residential county, baseline Charlson index. |
|  |  | HR = 1.16 (0.80, 1.68) | Baseline age, gender, years of education, occupation during most of life, smoking status, alcohol consumption status, physical activity level, neighbourhood socioeconomic status indicator, residential county, baseline Charlson index, baseline cognition function and primary language. |
| Havyarimana et al. (2025) (6) | Lden 24h   <50 dB 50-55 dB  55-60 dB  ≥ 60 dB | Ref  HR = 0.98 (0.85, 1.13)  HR = 1.12 (0.93, 1.33)  HR = 1.03 (0.84, 1.26) | Age, sex, individual-level SES (education, household income, current employment status), area-level SES, cardiovascular risk score, PM_2.5_, green space. |
| Tuffier et al. (2024) (7) | Lden per-IQR increase | HR = 1.10 (1.02, 1.17) | Calendar year. |
|  |  | HR = 1.08 (1.01, 1.16) | Calendar year, BMI, smoking, alcohol consumption, employment and marital status, family income. |
|  |  | HR = 1.07 (0.99, 1.16) | Calendar year, BMI, smoking, alcohol consumption, employment and marital status, family income, area level covariates (municipality type, median wealth, frequencies of unemployment, inhabitants receiving financial aid, and high education), PM_2.5_. |
|  |  | HR = 1.02 (0.93, 1.11) | Calendar year, BMI, smoking, alcohol consumption, employment and marital status, family income, area level covariates (municipality type, median wealth, frequencies of unemployment, inhabitants receiving financial aid, and high education), PM_2.5_. |
| Wu et al. (2024) (8) | For every 10dB increase in Lden | HR = 1.02 (0.89, 1.17) | Age, sex, education, baseline year, birth year. |
|  |  | HR = 1.00 (0.87, 1.15) | Age, sex, education, baseline year, birth year, SES, smoking, physical activity, number of medications, neighbourhood household mean income, hearing loss. |
|  |  | HR = 0.98 (0.82, 1.16) | Age, sex, education, baseline year, birth year, SES, smoking, physical activity, number of medications, neighbourhood household mean income, hearing loss, other environmental pollution (PM_2.5_, green space and water space). |

*Abbreviations: Leq 24h = equivalent continuous sound level over 24 hours; dB = decibels; HR = hazard ratio; CI = confidence intervals; BMI = body mass index; ApoE4 = Apolipoprotein E4; Lnightmax = Lnight exposure at most exposed façade of residence; Lnightmin = Lnight exposure at least exposed façade of residence; Lnight = A-weighted, equivalent noise level at night; IMD = Indices of Multiple Deprivation; iSES = individual socioeconomic status; nSES = neighbourhood socioeconomic status.*

**References**

1. Andersson J, Oudin A, Sundström A, Forsberg B, Adolfsson R, Nordin M. Road traffic noise, air pollution, and risk of dementia - results from the Betula project. Environ Res. 2018;166:334-9.

2. Cantuaria ML, Waldorff FB, Wermuth L, Pedersen ER, Poulsen AH, Thacher JD, et al. Residential exposure to transportation noise in Denmark and incidence of dementia: national cohort study. BMJ. 2021;374:n1954.

3. Carey IM, Anderson HR, Atkinson RW, Beevers SD, Cook DG, Strachan DP, et al. Are noise and air pollution related to the incidence of dementia? A cohort study in London, England. BMJ Open. 2018;8(9):e022404.

4. Ogurtsova K, Soppa VJ, Weimar C, Jöckel K-H, Jokisch M, Hoffmann B. Association of long-term air pollution and ambient noise with cognitive decline in the Heinz Nixdorf Recall study. Environmental Pollution. 2023;331:121898.

5. Yu Y, Mayeda ER, Paul KC, Lee E, Jerrett M, Su J, et al. Traffic-related Noise Exposure and Late-life Dementia and Cognitive Impairment in Mexican-Americans. Epidemiology. 2020;31(6):771-8.

6. Havyarimana E, Gong X, Jephcote C, Johnson S, Suri S, Xie W, et al. Residential exposure to road and railway traffic noise and incidence of dementia: The UK Biobank cohort study. Environmental Research. 2025;279:121787.

7. Tuffier S, Zhang J, Bergmann M, So R, Napolitano GM, Cole-Hunter T, et al. Long-term exposure to air pollution and road traffic noise and incidence of dementia in the Danish Nurse Cohort. Alzheimer's & Dementia. 2024;20(6):4080-91.

8. Wu J, Grande G, Pyko A, Laukka EJ, Pershagen G, Ögren M, et al. Long-term exposure to transportation noise in relation to global cognitive decline and cognitive impairment: Results from a Swedish longitudinal cohort. Environment International. 2024;185:108572.
